# Rhizosphere Bacterium *Rhodococcus* sp. P1Y Metabolizes Abscisic Acid to Form Dehydrovomifoliol

**DOI:** 10.3390/biom11030345

**Published:** 2021-02-25

**Authors:** Oleg S. Yuzikhin, Natalia E. Gogoleva, Alexander I. Shaposhnikov, Tatyana A. Konnova, Elena V. Osipova, Darya S. Syrova, Elena A. Ermakova, Valerii P. Shevchenko, Igor Yu. Nagaev, Konstantin V. Shevchenko, Nikolay F. Myasoedov, Vera I. Safronova, Alexey L. Shavarda, Anton A. Nizhnikov, Andrey A. Belimov, Yuri V. Gogolev

**Affiliations:** 1 All-Russia Research Institute for Agricultural Microbiology, Podbelskogo sh. 3, Pushkin, 196608 Saint-Petersburg, Russia; yuzikhin@gmail.com (O.S.Y.); ai-shaposhnikov@mail.ru (A.I.S.); syr_daria@ro.ru (D.S.S.); v.safronova@rambler.ru (V.I.S.); a.nizhnikov@arriam.ru (A.A.N.); belimov@rambler.ru (A.A.B.); 2 All-Russian Research Institute of Plant Protection, Podbelskogo sh. 3, Pushkin, 196608 Saint-Petersburg, Russia; 3 Kazan Institute of Biochemistry and Biophysics, Federal Research Center “Kazan Scientific Center of the RAS”, Lobachevsky Street, 2/31, 420111 Kazan, Tatarstan Republic, Russia; negogoleva@gmail.com (N.E.G.); eva-0@mail.ru (E.V.O.); ermakova@kibb.knc.ru (E.A.E.); 4 Kazan Federal University Institute of Fundamental Medicine and Biology, K.Marx, 76, 420012 Kazan, Russia; 5 Laboratory of Plant Infectious Diseases, FRC Kazan Scientific Center of RAS, 420111 Kazan, Tatarstan Republic, Russia; tatiana.a.konnova@gmail.com; 6 Institute of Molecular Genetics, Russian Academy of Sciences, Akademika Kurchatova Square, 2, 123182 Moscow, Russia; ATCarma@mail.ru (V.P.S.); nagaev@img.ras.ru (I.Y.N.); ATRegister@mail.ru (K.V.S.); nfm@img.ras.ru (N.F.M.); 7 Department of Genetics and Biotechnology, Saint-Petersburg State University, University Embankment, 199034 Saint-Petersburg, Russia; shavarda@binran.ru

**Keywords:** abscisic acid, microbial metabolite, dehydrovomifoliol, NMR spectrometry, phytohormones, rhizosphere, *Rhodococcus*

## Abstract

The phytohormone abscisic acid (ABA) plays an important role in plant growth and in response to abiotic stress factors. At the same time, its accumulation in soil can negatively affect seed germination, inhibit root growth and increase plant sensitivity to pathogens. ABA is an inert compound resistant to spontaneous hydrolysis and its biological transformation is scarcely understood. Recently, the strain *Rhodococcus* sp. P1Y was described as a rhizosphere bacterium assimilating ABA as a sole carbon source in batch culture and affecting ABA concentrations in plant roots. In this work, the intermediate product of ABA decomposition by this bacterium was isolated and purified by preparative HPLC techniques. Proof that this compound belongs to ABA derivatives was carried out by measuring the molar radioactivity of the conversion products of this phytohormone labeled with tritium. The chemical structure of this compound was determined by instrumental techniques including high-resolution mass spectrometry, NMR spectrometry, FTIR and UV spectroscopies. As a result, the metabolite was identified as (*4RS*)-4-hydroxy-3,5,5-trimethyl-4-[(*E*)-3-oxobut-1-enyl]cyclohex-2-en-1-one (dehydrovomifoliol). Based on the data obtained, it was concluded that the pathway of bacterial degradation and assimilation of ABA begins with a gradual shortening of the acyl part of the molecule.

## 1. Introduction

Abscisic acid (ABA) controls numerous aspects of the plant life cycle, including seed dormancy, germination and adaptive responses to environmental stresses [1]. The content of ABA in plant tissues is determined by the balance of its biosynthesis and catabolism. In plant catabolism, ABA does not undergo deep degradation, but is converted to inactive forms by oxidation or conjugation reactions [2,3]. The oxidation is the main pathway of ABA inactivation in plants. The key reaction of this pathway is hydroxylation at C-8′ to produce 8′-hydroxy ABA. It has been shown that in *Arabidopsis thaliana,* this reaction is catalyzed by the CYP707A2 monooxygenase [4]. 8′-hydroxy ABA spontaneously isomerizes to phaseic acid (PA) [5]. Significant amounts of ABA and, apparently, the products of its catabolism are constantly introduced into soil via the decomposition of abscised shoot tissues and root turnover. It has been shown that ABA transporters located in root epidermal cells can efflux ABA and its concentration in the soil solution can gradually increase during the growing season [6].

Higher plants are not monopolists in the production of ABA. ABA has been found at low concentrations in a wide range of tested organisms such as bacteria, cyanobacteria, algae, bryophytes, fungi and lichens [2,7]. Although often referred to as a phytohormone, ABA is an effective regulator of stress responses and pathogen biology in plants, parasitic protozoans, sponges, hydroids, insects, and mammals [8]. As shown in *Apis mellifera*, in insects, ABA may be involved in wound healing, pesticide sensitivity, and cold resistance [9]. Studies in mammals have also demonstrated the capacity of mammalian cells to respond to exogenous ABA. Biological responses include wide-ranging effects on inflammation, cell proliferation and glucose tolerance [10]. Until recently it was well accepted that bacteria do not synthesize ABA. However, in 2007, ABA was found in isolated strains of endophytic bacteria in the roots of *Helianthus annuus* [11]. Then, the ability to synthesize ABA was found in several plant growth-promoting rhizobacteria (PGPR), including *Azospirillum lipoferum* [12], *Arthrobacter koreensis* [13], *Achromobacter xylosoxidans*, *Bacillus licheniformis*, *Bacillus pumilus* and *Brevibacterium halotolerans* [14]. Fungi produce relatively large amounts of ABA that could be released into the environment [15]. Although little is known about the role of ABA in the metabolism of fungi, fungal ABA may be of some importance for plants infected with ABA-producing phytopatogenic fungi, in mycorrhizal associations and lichens [7]. In particular, there is ample evidence that ABA production is a significant contributor to the virulence of fungal phytopathogens [16,17].

The accumulation of ABA in soil can have a variety of effects on the development, resistance, and fitness of plants. Particularly, ABA accumulation during water stress may play a role in maintaining plant water status and growth in drying soil [18,19]. On the other hand, exogenous ABA can inhibit or promote root growth of well-watered plants depending on the phytohormone concentration [20,21,22]. Exogenous ABA decreased the elongation rate in maize coleoptiles [23]. The addition of ABA to mature non-dormant seeds has been shown to inhibit their germination. This effect of ABA might be related to its natural function as an endogenous inhibitor of precocious germination during seed formation [24]. The application of exogenous ABA or the inhibition of ABA biosynthesis revealed that increased ABA levels correlated with an increased susceptibility of plants to fungal [25,26,27] and bacterial pathogens [28].

The negative effects of ABA accumulation should probably be offset by the processes of its conversion. Since it is assumed that plants can contribute to, but do not regulate, the ABA concentration in soil, the plant associated microorganisms can claim this role. This hypothesis is supported by evidence that PGPR influences the rhizosphere concentration of other phytohormones, such as auxins [17] and ethylene [29,30], through the utilization of hormones or their precursors as carbon and/or nitrogen sources. It was also shown that *Burkholderia phytofirmans* [31], *Pseudomonas putida* [32], and *Rhizobium* spp. [33] degraded indole-3-acetic acid. *Serratia proteamaculans* utilized the cytokinin *N^6^*-benzyladenine [34], *Azospirillum* spp. transformed gibberellins [35] and several *Pseudomonas* spp. strains catabolized salicylic acid [36,37]. Recently, strains of *Achromobacter* sp., *Burkholderia* sp. and *Pseudomonas* sp. utilizing both indole-3-acetic and salicylic acids were identified [38]. However, information about the biochemistry of ABA biosynthesis and metabolism is very limited [7,8,39]. Nevertheless, there are results of preliminary studies that indicate the possible existence of more than one pathway of ABA catabolism in bacteria. In one study, it was shown that the introduction of radioactive ABA into non-sterile soil led to a rapid decomposition of this compound to phaseic acid and dehydrophaseic acid [6]. Earlier, it was reported that a soil bacterium, phenotypically designated as *Corynebacterium* sp., growing on a medium with ABA (2 g L^−1^) and yeast extract (1 g L^−1^), accumulated a compound with spectral characteristics similar to those of dehydrovomifoliol [40]. Unfortunately, the exact chemical structure of this compound has not been established. No further information about this bacterium and its ecological role in plant-microbe interactions is available.

Recently, using a selective ABA-supplemented medium, two bacterial strains were isolated from the rhizosphere of rice (*Oryza sativa* L.) and assigned to *Rhodococcus* sp. P1Y and *Novosphingobium* sp. P6W [41]. Both strains could utilize ABA as a sole carbon source in batch culture and decrease ABA concentrations in tomato roots or leaves. Correlations between the effects of these bacteria on plant growth and ABA concentrations in planta suggested that ABA-metabolizing rhizobacteria may interact with plants via an ABA-dependent mechanism. Thus, the aforementioned study [42] highlighted the importance of studying these microorganisms and the metabolic pathways of microbial ABA degradation.

The present report aimed to isolate and identify the chemical structure of an intermediate metabolite of ABA degradation by *Rhodococcus* sp. P1Y by a selection of techniques including high-resolution mass spectrometry, NMR spectrometry, FTIR and UV spectroscopies. The pool of data obtained thereby unambiguously indicate that this metabolite is dehydrovomifoliol.

## 2. Materials and Methods

### 2.1. Labeling ABA with Tritium

ABA was purchased from Merck (Sigma-Aldrich, St. Louis, MO, USA). A method for tritium labeling of the cyclohexene part of the ABA molecule has been reported [42]. Briefly, the reaction tube was charged with PdO (60 mg), 5% Pd/BaSO_4_ (15 mg) and anhydrous dioxane (0.3 mL). The tube was cooled with liquid nitrogen and evacuated down to 0.1 Pa, and tritium was injected up to 400 hPa pressure. Palladium oxide was reduced for 60–90 min at room temperature with stirring. The content of the tube was frozen in liquid nitrogen and evacuated down to 0.1 Pa to remove tritium. Dioxane and tritiated water were distilled into a tube containing 3 mg of ABA. The tube was filled with argon, diisopropylethylamine was added (4:1), and the reaction was conducted for 20 min at 220 °C. The reaction mixture was frozen in liquid nitrogen and volatile components were evaporated. The dry residue was dissolved in methanol. Labile tritium was removed by five times evaporation with methanol (3 mL). The residue was purified by HPLC on a Kromasil 100 C18 packed column (Eka Chemicals AB, Sweden, 8 × 150 mm size, 7 μm particle size) in a methanol:water:acetic acid solvent system (40:60:0.1) at a flow rate of 2 mL/min. The radiochemical purity of the product was analyzed on a Reprosil pur C18aq column (Dr. Maisch GmbH, Germany, 4 × 150 mm size, 5 μm particle size) using the following eluents: A, methanol:water:acetic acid (30:70:0.1); B, methanol; linear gradient from 0 to 50% of B in 15 min. The flow rate was 1 mL min^−1^. The retention time was 5.3 min. The yield of ABA was 50% and the specific radioactivity was 30.5 Ci mmol^−1^. The radiochemical purity of the final product was 98.5%.

### 2.2. Bacteria Cultivation

The bacterial culture cells were grown at 24 °C with shaking (200 rpm) in 100 mL of a mineral salt medium containing (g L^−1^): 14-Na_2_HPO_4_·12H_2_O, 3.0-KH_2_PO_4_, 1.0-NH_4_Cl, 0.3-MgSO_4_, 0.1-CaCl_2_, pH = 6.5. The medium was supplemented with 25 mg of ABA (250 mg L^−1^). When the cell culture absorbance reached 0.4 AU, an additional 25 of mg ABA and 50 μCi of labeled ABA was added. After 2 h of incubation, the bacterial suspension was centrifuged, and the supernatant was lyophilized.

Similar growth conditions and the medium were used to monitor the growth and utilization of putative low-molecular-weight products of the bacterial ABA metabolism, such as acetic, glycolic, glyoxylic and oxalic acids (Sigma-Aldrich). Bacterial growth was monitored daily by measuring the optical density of suspensions at 540 nm using a SmartSpec Plus spectrophotometer (Bio-Rad Laboratories Inc., Hercules, CA, USA). ABA and fructose were used as positive controls.

### 2.3. HPLC-MS Analysis Conditions

The lyophilized supernatant was extracted three times with 50 mL portions of methanol using an ultrasonic bath. The combined extract was evaporated to dryness on a rotary evaporator at 40 °C. As a result, 34.1 mg of dry residue was obtained. The residue was dissolved in 10 mL of methanol and analyzed by HPLC-MS using a 6538 Q-TOF mass spectrometer (Agilent Technologies, Santa Clara, CA, USA) equipped with an ESI interface. A Waters BEH-C18 column (100 × 3.0 mm, 3.5 μm) was used. Chromatography was performed at ambient temperature with an injection volume of 5 µL. The flow rate was 0.15 mL/min. The mobile phases were water–acetonitrile 95:5 + 0.1% formic acid (A) and acetonitrile–water 90:10 + 0.1% formic acid (B). The gradient was as follows: 0–1 min 0% B, 1–13 min 0% → 90% B; 13–20 min, 90% B; and 20–25 min, 90% → 0% B. The Q-TOF/MS analysis was carried out in full-scan mode, and the mass range was m/z 100−2000 in the negative and positive modes, with a scan rate 2 spectra/sec. The operating parameters of the electrospray ionization sources were as follows: drying gas (N_2_) flow rate, 7.0 L/min; drying gas temperature, 350 °C; nebulizer pressure, 30 psig; fragmentor voltage, 175 V; capillary, 3500 V; octapoleRFPeak, 750 V; and skimmer voltage, 65 V. All the acquisitions and analyses of data were controlled by MassHunter software (version B.05.00, Agilent Technologies, Santa-Clara, CA, USA). A calibrating solution (calibrant solution A, Agilent Technologies, Santa-Clara, CA, USA) containing internal reference masses at m/z 119.0363 and 966.0007 in negative and 121.050873 and 922.009798 in positive ion mode was used in conjunction with an automated calibration. Parallel UV detection was performed using an Agilent 1260 Series G1365D Multiple Wavelength Detector instrument.

### 2.4. Isolation of Metabolite I

The preparative chromatographic separation of the metabolites was performed on silica gel (Merck 60) using the MPLC system Buchi Sepacore and completed with the UV-Monitor C-630, two Pump Module C-605, Control Unit S-620, fractions collector C-660 (Buchi, Flawil, Switzerland). The solvents were (A) n-hexane, (B) EtOAc, and (C) MeOH. The methanol solution (obtained as described in Section 2.3) was loaded onto 5 mL of silica gel, blowing off methanol in a stream of nitrogen. The resulting material was introduced in hexane into a cartridge, which was connected in series with a Glass Column Buchi 15/230 (40.65 mL) filled with the same silica gel and preconditioned in a hexane flow (15 mL min^−1^ for 5 min). The separation conditions are shown in Table 1. Eluent flow rate was 15 mL min^−1^. Fractions of 45 mL were collected and analyzed by liquid scintillation counting using a QuantaSmart Tri-Carb 2810TR instrument (PerkingElmer, Inc., Waltham, MA, USA). The purity and composition of the obtained fractions were estimated by the UPLC method using an Acquity H-class chromatograph with PDA detector. A Waters ACQUITY UPLC BEH C18 column (50 × 2.1 mm, 1.7 μm) was used for analysis. Chromatography was performed at 30 °C. The injection volume was 5 µL. The flow rate was 0.3 mL min^−1^. The mobile phases were water with 0.1% formic acid (A) and acetonitrile with 0.1% formic acid (B). The gradient was as follows: 0–1 min 0% B, 1–13 min 0% → 90% B; 13–14.1 min 90% → 0% B. The detection wavelength was 265 nm. MPLC fractions 10 and 11, which showed high radioactivity and contained (as shown by UPLC analysis) one major component, were combined and evaporated to dryness at 40 °C using a vacuum rotary evaporator Heidolph Hei-VAP Precision (Heidolph Instruments GMBH and CO KG, Schwabach, Germany). The dry residue weight was 2.1 mg (6.2% yield).

The final purification of the compounds was carried out by preparative HPLC using a Waters 2545 HPLC completed with a 2489 UV/VIS detector, a Waters XBridge Prep C18 OBD column (250 × 19 mm, 5 µm) and a fraction collector III (Waters Corporation, Milford, MA, USA). Chromatography was performed at ambient temperature. Before the chromatographic separation, the dry residue obtained after MPLC separation was dissolved in 5 mL of methanol. Injection volume was 1 mL. The flow rate was 5 mL min^−1^. The mobile phases were water (A) and acetonitrile (B). The gradient was as follows: 0–1.5 min 0% B, 1.5–13.5 min 0% → 90% B; 13.5–15 min, 90% B; and 15–16 min, 90% → 0% B. The detection wavelength was 265 nm. Under the conditions of chromatography, the collection of ABA was carried out in the time interval of 8.2–9.0 min, and the target metabolite, in the interval of 7.2–7.7 min. The final solutions were pools of the corresponding fractions after five successive separations.

After the evaporation of the solvent, 1.3 mg (yield 3.8%) of a chromatographically pure compound was obtained, which was analyzed by liquid scintillation counting and spectrometric analyses.

### 2.5. Spectrometric Analyses

A UV spectrum of metabolite I was recorded in the HPLC mode using a diode array detector. The spectrum was obtained in the solvent system water–acetonitrile (32%) with 0.1% formic acid, analytical flow cell with a volume of 500 nL and path length of 10 mm.

One- (^1^H, WET1D) and two-dimensional (HSQC, HMBC, H2BC) nuclear magnetic resonance (NMR) spectra were recorded on a DirectDrive NMR System (Varian, Palo Aho, CA, USA) in CDCl_3_ at 700 and/or 175.8 MHz. CDCl_3_ was used as an internal standard.

For the FTIR spectroscopic measurements, 1 mg of the resulting substance was dissolved in 10 μL of methanol (99.9%, Acros Organics, Antwerp, Belgium), placed with a microsyringe as a thin film on clean flat CVD-ZnSe disks with the diameter of 10 mm and thickness of 2 mm (R’AIN Optics, Dzerzhinsk, Russia) and dried. Transmission FTIR spectroscopic measurements were performed on a Nicolet 6700 FTIR spectrometer equipped with a DTGS detector and a KBr beam splitter (Thermo Electron Corporation, Beverly, MA, USA). Spectra were collected with a total of 64 scans (resolution 4 cm^–1^) against the CVD-ZnSe disc background and manipulated using the OMNIC software (version 8.2.0.387, Thermo Fisher Scientific, Waltham, MA, USA) supplied by the manufacturer of the spectrometer. The baseline was corrected using the “automatic baseline correct” function. The spectra were smoothed using the standard “automatic smooth” function of the software which uses the Savitsky–Golay algorithm (95-point moving second-degree polynomial). The FTIR spectroscopic measurements were repeated three times and were well reproducible.

### 2.6. Molecular Geometry Calculations

The geometry optimization of two enantiomers was performed by us using the GAUSSIAN program (version GAUSSIAN09, Gaussian, Inc., Wallingford, CT, USA) and the DFT method at the b3lyp/6-311+G(d,p) level of theory.

### 2.7. Statistical Analysis

The statistical analysis of the data presented in Figure 7 was performed using the software STATISTICA version 10 (TIBCO Software Inc., Palo Alto, CA, USA).

## 3. Results

### 3.1. Identification and Isolation of Metabolite I

**Figure 1:**
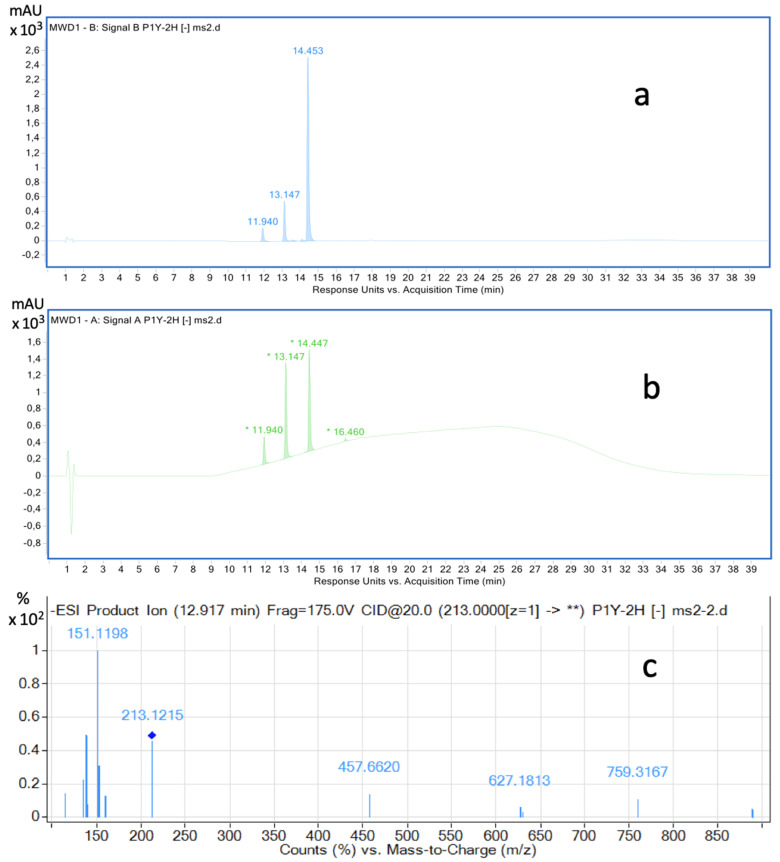
Chromatograms of the components of the *Rhodococcus* sp. P1Y culture liquid 2 h after adding the tritium labeled ABA. HPLC-MS, registration at two wavelengths: 254 nm (**a**) and 220 nm (**b**). Mass spectra of components: ESI–TOF MS, negative ion mode (**c**), E_col_ = 20 V m/z (relative intensity): compound 14.4, abscisic acid (ABA): 263.1291 (C_15_H_19_O_4_, calcd. 263.1283) [M-H]^−^ (100), 219.1392 (C_14_H_19_O_2_, calcd. 219.1385) [M-H-CO_2_]^−^ (14); Compound 13.1 (metabolite I) (**c**): 213.1203 (C_11_H_17_O_4_, calcd. 213.1127) [M + 2H_2_O- H-CH_3_CHO]^−^ (45), 153.0951 (C_9_H_13_O_2_, calcd. 153.0916) (31), 151.1198 (C_10_H_15_O, calcd. 151.1123) (100), 138.0747 (C_8_H_10_O_2_, calcd. 138.0681) (49), 135.0874 (C_9_H_11_O, calcd. 135.0810) (22); compound 11.9: 212.1002 (^13^C- Isotope ion, calcd. 212.1004) [M-H + 1]^−^ (12), 211.0969 (C_11_H_15_O_4_, calcd. 211.0970) [M-H]^−^ (100), 167.1077 (C_10_H_15_O_2_, calcd. 167.1072) [M-H-CO_2_]^−^ (41), 152.0834 (C_9_H_12_O_2_, calcd. 152.0837) [M-CH_3_COOH]^−^ (8), 149.0972 (C_10_H_13_O, calcd. 149.0966) [M-H-CO_2_- H_2_O]^−^ (9).

Strain *Rhodococcus* sp. P1Y grew well on the mineral medium containing ABA as a sole carbon source. Analysis of the culture fluid by the HPLC-MS revealed two major and several minor compounds, presumably microbial metabolites of this phytohormone (Figure 1). The present research focused on the purification and identification of the main compound, named metabolite I.

Experiments with a radioactive precursor confirmed that the detected compounds originate from ABA. When the microorganism was grown on 25 mg of labeled ABA (2384 cpm/µg, 284 mCi/mol), the radioactive substrate accumulated in two fractions of preparative chromatographic separation (Table 1). These fractions were re-separated in an acetonitrile–water system. After the evaporation of the solvent, chromatographic purification gave 1.3 mg of material (yield 3.8%) as a colorless oil. The purity of the isolated compound was established by the UPLC as described in the Materials and Methods (data not shown). The radioactivity of the obtained metabolite was 2562 cpm μg^−1^, which is 263 mCi mol^−1^ (see below). This value corresponds well to the activity of ABA as a precursor.

### 3.2. Chromatography-Mass Spectrometric Measurements

**Figure 2:**
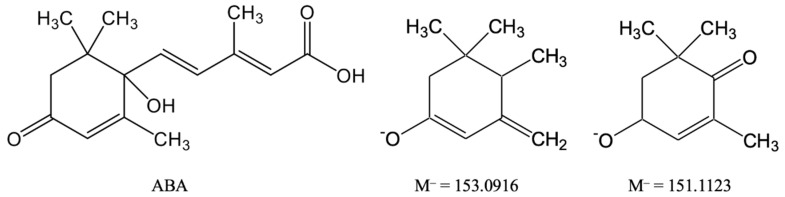
Calculated structures of the main ions obtained by ESI-TOF MS in negative ionization mode.

ESI-TOF MS analysis in negative ionization mode has been performed for ABA and Compound I in HPLC-MS analysis of culture liquid components (Figure 1c). In this case, the molecular ion of metabolite I was not obtained. The main ions were ions with masses M^–^ = 153.0916 and 151.1123. The calculated structures of these ions are shown in Figure 2. These ions were obtained as a result of the detachment of the side chain of the ABA molecule, which indicates the intactness of the cyclohexene residue in metabolite I. The absence of a molecular ion in the spectra may be due to its instability and the absence of strong acid groups in the metabolite structure.

**Figure 3:**
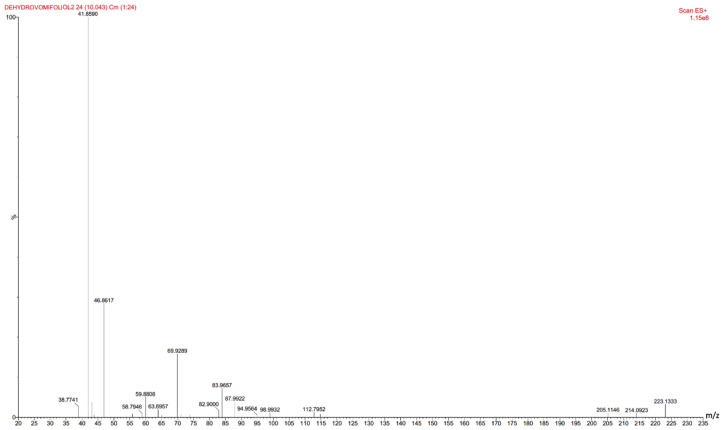
Mass spectrum of metabolite I obtained in the positive electrospray ionization mode. ESI–TOF MS *m*/*z* (relative intensity): 223.1333 (C_13_H_18_O_3_, calcd 223.1334) [M + H]^+^ (3), 205.1225 (C_13_H_17_O_2_, calcd 205.1229) [M + H-H_2_O]^+^ (1), 87.9922 (C_3_H_4_O_3_, calcd 88.0160) (4), 69.9989 (C_3_H_2_O_2_, calcd 70.0055) (16), 46.8617 (CH_3_O_2_, calcd 47.0133) (28), 41.8590 (C_2_H_2_O, calcd 42.0106) (100).

Mass spectra for the purified metabolite I were obtained in the positive electrospray ionization mode (Figure 3). The spectrum contained the molecular ion M^+^ 223.1334, as well as the M^+^ 205.1225 ion. The latter can be obtained as a result of the abstraction of a water molecule, which is typical for alcohols. However, the main ions were ions of deep fragmentation, probably associated with breaks in the cyclohexene part of the molecule.

### 3.3. NMR Spectroscopy

A detailed identification of the chemical structure of metabolite I was carried out using NMR methods, including one-dimensional (1H, WET1D) and two-dimensional (HSQC, HMBC, H2BC) experiments. The HSQC spectrum made it possible to establish the binding of carbon atoms with protons. Long-range carbon–proton interactions were obtained from the HMBC and H2BC spectra, which made it possible to establish the carbon skeleton of the molecule. A ^13^C NMR spectrum of metabolite I was extracted from the corresponding 2D spectra. Below are the spectral characteristics and their corresponding interpretation. ^1^H NMR: 6.83 (1H, d, J = 15.8 Hz, H-1′), 6.46 (1H, d, J = 15.7 Hz, H-2′), 5.96 (1H, dd, J = 1.2, 1.0 Hz, H-2), 2.50 (1H, dm, J = 17.2 Hz, H-6a), 2.34 (1H, dm, J = 17.1 Hz, H-6b), 2.30 (3H, s, H-4′), 1.88 (3H, d, J = 1.3 Hz H-5′), 1.82 (1H, d, J = 0.3 Hz, OH), 1.11 (3H, s, H-6′), 1.01 (3H, s, H-7′). ^13^C NMR: 199.9 (C-3′, C=O), 199.6 (C-1, C=O), 162.7 (C-3, Cq), 147.3 (C-1′, HC=CH), 132.9 (C-2′, HC=CH), 130.3 (C-2, HC=C), 81.8 (C-4, C–OH), 52.1 (C-6, CH_2_), 44.0 (C-5, Cq), 30.8 (C-4′, CH_3_), 26.8 (C-7′, CH_3_), 25.4 (C-6′, CH_3_), 21.1 (C-5′, CH_3_). UV (λ_max_): 239.0 nm. More detailed information is given in the Supplementary File S1. The ABA spectra (Supplementary File S2) were obtained under the same conditions of the NMR experiment that were used for the studied metabolite I.

### 3.4. Optical Spectroscopy

**Figure 4:**
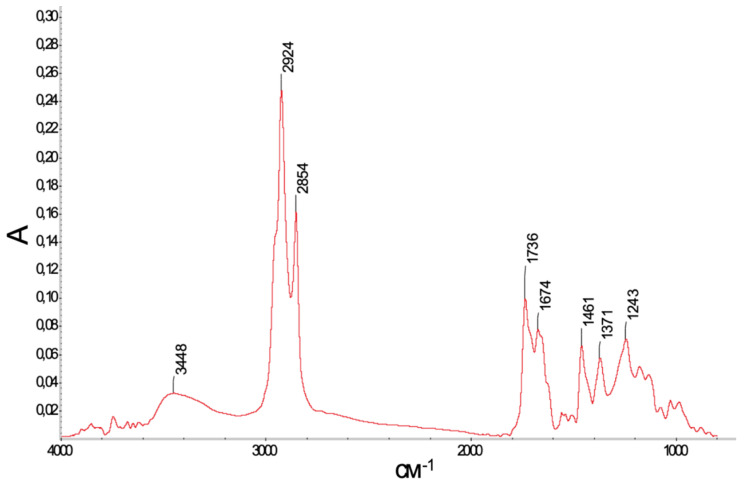
A transmission FTIR spectrum of metabolite I. IR (ν_max_, cm^−1^; band shape and vibration type assignments are given in parentheses): 3448 (broad; ν OH); 3000 (shoulder, weak; ν =C–H); 2955 (shoulder, strong), 2924, 2854 (ν_as_ CH_3_, ν_as_ CH_2_, ν_s_ CH_3_, ν_s_ CH_2_); 1736 (asymmetric, broad; ν C=O), 1674 (asymmetric, broad; ν C=C), 1461 (asymmetric; δ_as_ CH_3_, scissoring δ CH_2_), 1371 (δ_s_ CH_3_), 1243 (broad; ν C–CH_3_, C–C).

A transmission FTIR spectrum of metabolite I is shown in Figure 4.

An UV spectrum of metabolite I was recorded in the HPLC experiment. This spectrum has one peak with an absorption maximum at 239 nm (Figure S3).

## 4. Discussion

The comparison of the spectra of ABA and metabolite I made it easier to identify the structure of the latter. In addition, this made it possible to clarify the spectral characteristics of ABA previously published (https://www.chemicalbook.com/CASEN_21293-29-8.htm#SpectrumDetail, accessed 12 December 2020). Basically, the spectra obtained here for ABA were identical to spectra obtained earlier, with the exception of the assignment of signals from protons 3 and 2. The assignment of the given signals to the corresponding protons made previously should be swapped, which follows from the analysis of our two-dimensional spectra. The same should be carried out with the signals from protons four and five. All chemical shifts of ^13^C atoms in the given spectrum are by 2–3 ppm less than in the spectrum obtained in the present study. This can be explained by the difference in the anchoring of the reference peak (in our case, the CHCl_3_ peak).

Despite the large amount of material (25 mg) taken for the ^13^C NMR spectrum, we were unable to detect the peaks of the C-1 and C-1’ quaternary carbon atoms. The position of these atoms was established by analyzing the ^13^C-^1^H long-range spin-spin interactions in the HMBC spectrum.

The comparison of the NMR spectra of ABA and metabolite I showed their identity in the cyclohexenone part of the molecule, including the methyl substituents. This confirms the assumption made on the basis of mass spectrometry that the biodegradation of the ABA molecule begins from the side chain. In turn, the structure of the side chain of metabolite I can be obtained by analyzing one- and two-dimensional NMR spectra. In contrast to ABA, the double bond of the side chain of metabolite I is conjugated not with the next double bond, but with the carbonyl group, which is confirmed by the displacement of the chemical shifts of the corresponding carbon atoms toward a weaker field.

**Figure 5:**
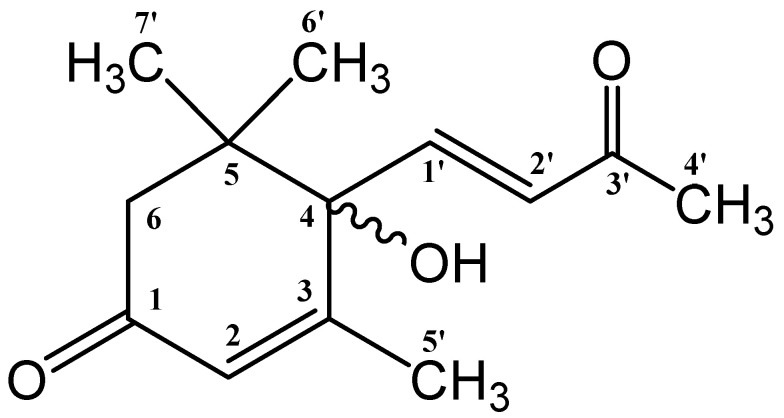
Structure of dehydrovomifoliol obtained from the results of the analysis of NMR and mass spectra.

Taken together, the obtained spectral characteristics coincided with the characteristics of dehydrovomifoliol, (*4RS*)-4-hydroxy-3,5,5-trimethyl-4-[(*E*)-3-oxobut-1-enyl]cyclohex-2-en-1-one), published earlier [43,44]. The structure of this compound is shown in Figure 5.

The interpretation of the FTIR spectrum of the obtained metabolite I (Figure 4) deserves special attention, as it provides additional information confirming the structure of this compound. Modern FTIR spectroscopy has been increasingly applied in microbiology-related studies for both qualitative (structural) and quantitative bioanalyses (see, e.g., [45,46,47]). In addition, the data available on IR studies of dehydrovomifoliol cannot be considered as exhaustive. Takasugi et al. [48] indicated only four bands (by their maxima only) in a spectrum measured in CHCl_3_. In that case, the authors of [48] did not indicate the concentration of the solvent, which makes it difficult to assess its possible role in shifting the band maxima. Häusler et al. [49] gave more spectroscopic bands (not presenting the spectrum either) measured in CCl_4_. The comparison of the published infrared spectroscopic data with those obtained in this work (for the purified substance without a solvent) shows their general similarity.

The differences in the positions of the bands are evidently caused by the influence of the solvents chosen for the measurements in [48,49]. The authors of [48,49] also did not indicate the concentrations of dehydrovomifoliol. However, taking into account the shift of the maximum of the broad stretching vibration band of the OH group (3580 cm^–1^ as indicated in [49]) to the lower frequency region (~3448 cm^–1^; see Figure 4), it can be assumed that the intermolecular association of the OH groups in a CCl_4_ solution [49] is insignificant as compared to that in our spectrum (where the recording conditions were in the pure condensed phase). For the C–H stretching vibrations, Häusler at al. [49] gave only one band (at 2960 cm^–1^), which is probably due to the low resolution of their measuring equipment. However, since the dehydrovomifoliol molecule has four methyl groups and one methylene group, each of them should give two different bands of asymmetric and symmetric stretching vibrations within the region 3000–2800 cm^–1^. In addition, there are three =CH moieties (which usually give weak FTIR stretching C–H bands near or slightly above 3000 cm^–1^). Thus, the spectrum obtained in this work with strong and clearly asymmetric (i.e., composite) bands at 2924 and 2854 cm^–1^ is more informative reflecting the structure of the title compound. 

In the spectrum of dehydrovomifoliol in the region 1600–1750 cm^–1^, there should be at least four neighboring bands: two strong, corresponding to the different carbonyl groups (see Figure 5), and two weak, corresponding to stretching vibrations of the C=C bonds. As described earlier (Bellamy, p. 55, Table 2 [50]), conjugation of the C=C bond with the carbonyl group increases the intensity of stretching C=C vibrations. In addition, the effect of the substituent (the OH group), which is in the allyl position to both double bonds, is similar to the effect of halogens and is expected to shift the bands of stretching C=O and C=C vibrations to higher frequencies.

Indeed, in the obtained spectrum (see Figure 4), two bands of comparable intensities can be noted at 1736 and 1674 cm^–1^. The first of them evidently corresponds to the stretching vibrations of the C=O groups, the second to the C=C bonds. Both bands exhibit asymmetry, which is explained by a superposition of two neighboring bands of structurally nonequivalent carbonyl groups and C=C moieties (see Figure 5) in each of the two bands. For the band at 1371 cm^–1^, its δ_s_ assignment is typical. In Bellamy’s monograph [50], it is indicated for C–CH_3_ and C(CH_3_)_2_ (with the possibility of two bands) in the narrow range 1365–1385 cm^–1^. In the spectrum obtained here, there is a band in this region with a noticeable broadening.

**Figure 6:**
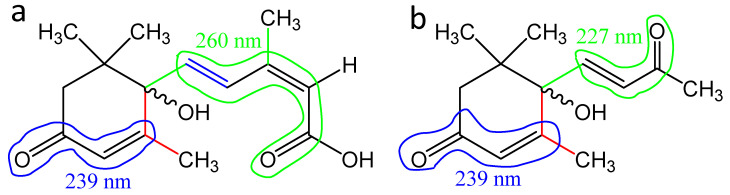
Systems of double bonds in ABA (**a**) and dehydrovomipholiol (**b**) which determine the base value of λ_0_ for calculating the maximum UV absorption wavelength.

For the UV spectra of dehydrovomipholiol and ABA, we calculated the theoretical absorption wavelength maxima for the π → π* transitions according to Woodward’s empirical rules [51]. For a system of double bonds conjugated with a carboxyl group in ABA, this calculation leads to a value of 260 nm. In this case, the base value λ_0_ = 200 nm in the calculations was taken to be the absorption maximum in the spectrum of acrylic acid (Figure 6): λ_max_ = λ_0_ + Δλalk^β^ + Δλalk^δ^ + Δλ(C=C) = 200 + 12 +18 + 30 = 260 nm. This value correlates well with the experimental absorption maximum for ABA, which is in the range 263–265 nm, depending on the measuring conditions. The slight difference is possibly due to the fact that the carboxyl group is internally hydrogen bonded to the hydroxyl in the cycle.

According to the data presented above, the bacterial destruction of the ABA molecule begins from the side chain and affects one of the conjugated double bonds. This should lead to a shift in the absorption wavelength maximum of the π → π* transition to the short-wavelength region.

For dehydrovomifoliol, the calculated value of the absorption maximum is λ_max_ = λ_0_ + 2Δλalk^β^ = 215 + 2 × 12 = 239 nm, where λ_0_ = 215 nm is the value indicated for cyclic enones in six-membered cycles. The obtained value coincides with the absorption wavelength of the revealed ABA metabolite and may be an indirect confirmation of the found structure.

Until now, the role of microorganisms in maintaining ABA homeostasis in soil remains unclear. Apparently, bacteria capable of degrading ABA such as *Rhodococcus* sp. P1Y use this phytohormone as a sole source of carbon and energy [41]. The well-known pathway of ABA transformation is the modification of the cyclohexene part of the molecule with the formation of phaseic and dehydrophaseic acids, which is characteristic for plants and also proposed for soil microbial communities [6]. The report on the detection of dehydrovomifoliol as the main degradation product in the culture of soil bacterium *Corynebacterium* sp. [40] did not receive, in our opinion, due attention. In the present study, we demonstrated the ability of the rhizosphere bacterium *Rhodococcus* sp. P1Y to decompose ABA with the formation of dehydrovomifoliol when growing on an artificial nutrient medium containing this phytohormone as a sole carbon and energy source. It should be noted that the above-mentioned *Corynebacterium* sp. co-metabolized ABA in the presence of yeast extract in high concentration [40]. The presence of at least one more metabolite (metabolite II) in the culture liquid of *Rhodococcus* sp. P1Y having a lower molecular weight (Figure 1), indicates further decomposition of ABA in the course of an unknown metabolic pathway. This metabolite II is currently being studied.

**Figure 7:**
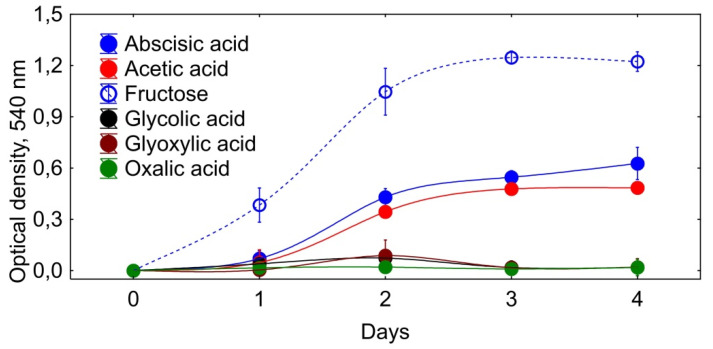
Growth of *Rhodococcus* sp. P1Y in a liquid mineral salt medium supplemented with different compounds as a sole carbon source. Carbon sources are shown in the figure. Vertical bars indicate confidence intervals (*n* = 3; *p* < 0.05).

There are currently no sufficient experimental data to describe a possible reaction mechanism for ABA conversion to dehydrovomifoliol. The presence of a branching point at position three of the ABA molecule may hinder normal β-oxidation. However, in the literature there are evidences for overcoming the branching point by soil bacteria. Accordingly, *Nocardia cyriacigeorgica* was able to shorten β-methylcinnamic acid due to the cleavage of the C2-unit with the formation of acetophenone [52]. As for *Rhodococcus* sp. P1Y, we can assume the elimination of the C2-unit in the reaction of meta-terminal oxidation by the mechanism proposed earlier for the degradation of alkenes by *Acinetobacter radioresistens* [53]. In this case, the second product of the ABA decomposition would be oxalic acid. However, we were unable to detect oxalic acid in the studied samples. The formation of glycolic or glyoxylic acids as a result of oxidation or acetic acid as a result of double bond cleavage can be considered as alternative options. However, our experiments with batch cultures showed that among these substrates, only acetate supported the growth of *Rhodococcus* sp. P1Y (Figure 7). Our genomic studies revealed an impressive arsenal of hydrolytic enzymes, as well as oxygenases, in ABA-utilizing bacteria *Rhodococcus* sp. P1Y [54] and *Novosphingobium* sp. P6W [55]. The precise establishment of the reaction mechanism, identification of the products of the subsequent dehydrovomifoliol degradation, and determination of the enzymes involved in these processes are now under study.

Another question that would be interesting to elucidate is the absolute configuration of the C-4 in dehydrovomifoliol formed by the bacterium. Since this would require X-ray crystallography which cannot be performed with a liquid sample, and the compound does not contain an atom with a high atomic weight, this task is quite problematic. The geometry optimization of two enantiomers, performed by us using the GAUSSIAN09 program and DFT calculations, has shown that the enantiomers have similar energies: the difference is less than 0.5 kcal/mol, which is within the limits of calculation accuracy and thus no reliable prediction of ORD (optical rotatory dispersion) or CD (circular dichroism) spectra can be made.

## 5. Conclusions

During the cultivation of the rhizosphere bacterium *Rhodococcus* sp. P1Y on the medium supplemented with ABA as a sole carbon source, two metabolites of this phytohormone were detected. A detailed characterization showed that the main metabolite is dehydrovomifoliol. The results obtained allow us to conclude that there is an uncharacterized pathway of ABA degradation by rhizosphere bacteria starting with the shortening of the acyclic part of the molecule.

## Figures and Tables

**Table 1:** Conditions for MPLC separation of metabolites and radioactivity of chromatographic fractions of the *Rhodococcus* sp. P1Y culture after incubation for 2 h in the presence of ^3^H-labeled ABA.

No. of Fraction	Starting Solvents Ratio, Vol %	Final Solvents Ratio, Vol %	Radioactivity, cpm µL^−1^
1	A 100	A-B 90:10	10.1 ^1^
2	A-B 90:10	A-B 80:20	7.0
3	A-B 80:20	A-B 70:30	2.2
4	A-B 70:30	A-B 60:40	2.5
5	A-B 60:40	A-B 50:50	1.0
6	A-B 50:50	A-B 40:60	-
7	A-B 40:60	A-B 30:70	-
8	A-B 30:70	A-B 20:80	-
9	A-B 20:80	A-B 10:90	3.5
10	A-B 10:90	B 100	34.5
11	B 100	B-C 50:50	168.2
12	B-C 50:50	C 100	164.3
13	C 100	C 100	5.6

^1^ The volume of each fraction was 45 mL.

## Data Availability

All relevant data are within the paper and its Supplementary Materials files.

## Supplementary Materials

The following are available online at https://www.mdpi.com/2218-273X/11/3/345/s1, Table S1. 1H and 13C NMR spectra of dehydrovomifoliol; Table S2. Comparison of experimental and published (Cb) 1H and 13C NMR spectra of abscisic acid; Figure S3. UV spectrum of dehydrovomifoliol.
