# Supplementary figures and images for: Rhizosphere Bacterium Rhodococcus sp. P1Y Metabolizes Abscisic Acid to Form Dehydrovomifoliol
Source: Biomolecules. 2021 Feb 25;11(3):345. doi: 10.3390/biom11030345 (PMC7996341; doi:10.3390/biom11030345)

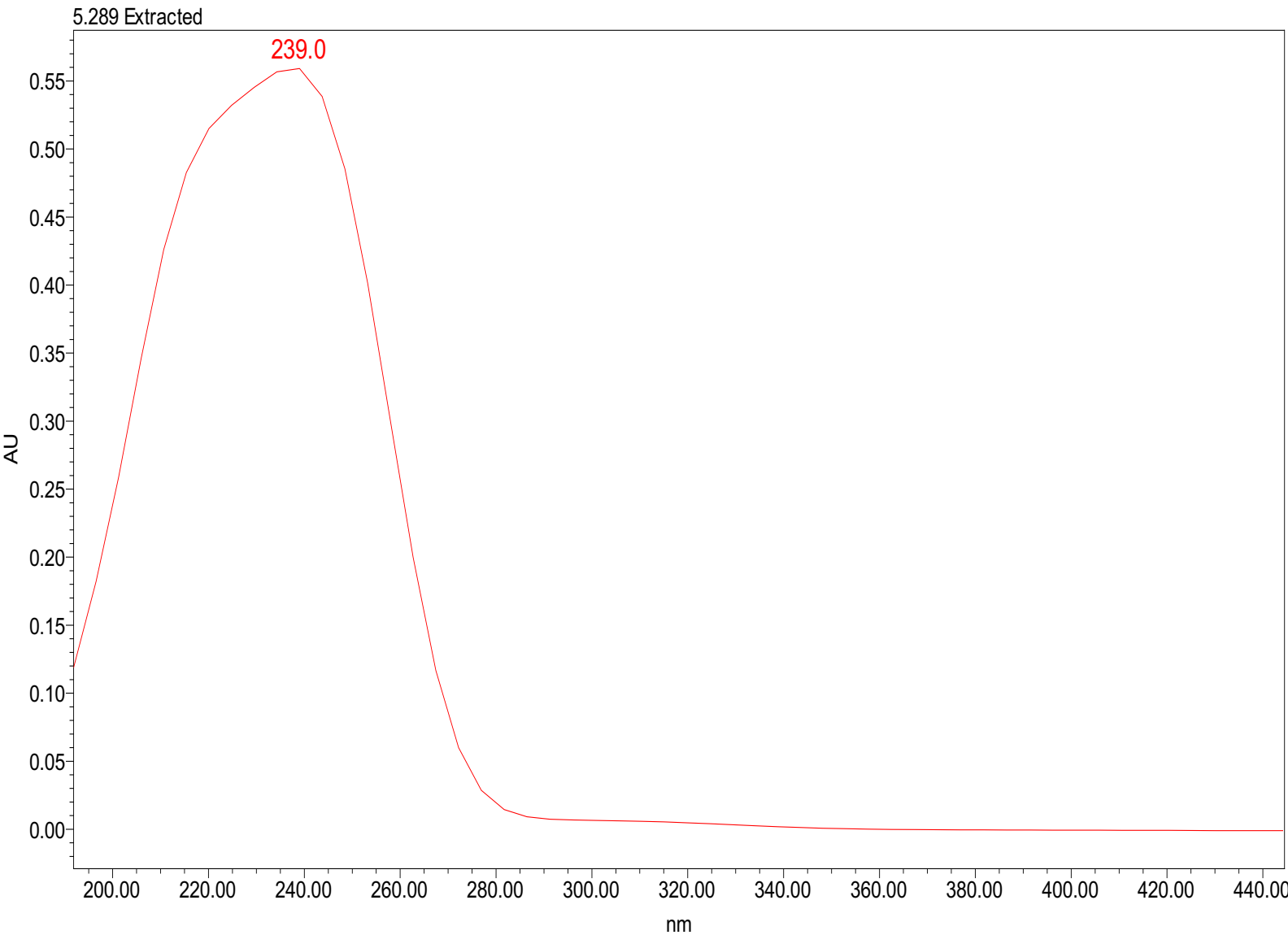

Supplement: Supplementary file 1 [file biomolecules-11-00345-s001.zip › biomolecules-1038940-supplementary/Fig S3. UV spectrum of dehydrovomifoliol.pdf]
